# Guest Editorial: Tenth Anniversary of the Journal of Applied Clinical Medical Physics

**DOI:** 10.1120/jacmp.v10i4.3196

**Published:** 2009-10-20

I am pleased to write this guest editorial on the occasion of the tenth anniversary of the Journal of Applied Clinical Medical Physics and I wish to thank the present Editor‐in‐Chief, George Starkschall, for the invitation to do so.

When we started ten years ago the idea of an online journal was quite new. Systems for handling an entirely electronic journal including submission, review, editing, and advertising had not been fully developed. Most journals that were online at that time were primarily print journals that were posted on the Internet. JACMP being a new journal had no print version. Our early partner was the American Institute of Physics Publishing who was, at that time, developing a system for online publishing. Their help and expertise were vital to the success of the journal and they went beyond the terms of their contract to help get the journal started. When the American College of Medical Physics (ACMP) asked me to undertake this task and to become the Editor‐in‐Chief, I knew nothing about electronic publishing and had more to learn than anyone else. But AIP Publishing proved a good mentor and Volume 1 Issue 1 went online just one‐and‐a‐half years after the College gave the initial go‐ahead.

Several criteria for the journal had been established by the ACMP: it was to be entirely electronic, there would be no print version; it was to be a free journal supported by advertisers; it was to be a journal devoted to the clinical applications of medical physics; its targeted readers and contributors were medical physicists worldwide; it aimed to have a short turnaround time from submission to publication – although this, of course, would depend upon how quickly referees submitted their reports and authors returned their revised manuscripts, and how user‐friendly the system was. That most of these aims have been met is a tribute to the Editors‐in‐Chief, the editorial boards, the advertisers, the referees, the authors and publishing partners who have served the journal over the last ten years. Vital to those initial four years was the manuscript manager and I am indebted to Georgeanne Moore who learned the electronic publishing business with me and then handled all the manuscripts while I was Editor‐in‐Chief. I was also fortunate to have an excellent editorial board that put in a lot of time and deserve much of the credit in getting the journal started.

The journal would not have been possible without the support and encouragement of the members of the American College of Medical Physics and, in particular, the vision of Michael Mills who campaigned early for a journal and who served as the third Editor‐in‐Chief. Getting the journal started was not inexpensive and the College made the commitment to underwrite the cost (including, I am thankful to acknowledge, my financial support).

As a reminder of those early years, I keep in my office printed copies of the first three volumes of the journal. In Volume 1 we averaged slightly less than five papers per issue. As I write this editorial, the last four issues have averaged nearly 15 papers (including technical reports, book reviews, letters to the editor, etc.) per issue. This represents a three‐fold increase in 10 years, and the trend continues upward. The journal is well and going strong.

The mission statement of the journal was published in the first issue and states: “…to publish papers that will help clinical medical physicists perform their responsibilities more effectively and efficiently for the increased benefit of the patient.” And this statement still appears on every issue. A quick review of the papers published over these last ten years shows that it has lived up to this expectation. And it has truly become an international journal with papers being published from around the world (over 20 countries) and – it can be concluded – read worldwide.

Each year our first and second year medical physics’ students are required to present a twenty‐minute summary of a published paper that they find interesting and believe would be of interest to their fellow students. Increasingly they are choosing articles published in JACMP.

It appears that it is firmly established in the list of medical physics journals that should be read by those entering the field.

Congratulations to the present Editor‐in‐Chief, my friend and colleague George Starkschall. I wish him and the journal continuing success in the coming years.

Peter Almond, Ph.D

